# Targeting PRC2: RNA offers new opportunities

**DOI:** 10.18632/oncotarget.22715

**Published:** 2017-11-27

**Authors:** Xueyin Wang, Chen Davidovich

**Affiliations:** Department of Biochemistry and Molecular Biology, Monash University, Clayton, VIC 3800, Australia; Biomedicine Discovery Institute, Faculty of Medicine, Nursing and Health Sciences, Monash University, Clayton, Australia; EMBL-Australia and the ARC Centre of Excellence in Advanced Molecular Imaging, Clayton, Australia

**Keywords:** polycomb repressive complex 2, PRC2, RNA, G-quadruplex, anticancer therapeutics

The histone methyltransferase Polycomb repressive complex 2 (PRC2) is essential for embryonic development and is dysregulated in multiple types of cancer [1, 2]. PRC2 methylates lysine 27 of histone H3, to produce the H3K27me3 histone modification of repressed chromatin. Thus, PRC2 keeps thousands of genes in an off state during development. It does so through cooperation with multiple factors, including accessory proteins, specific histone marks, DNA elements and RNA transcripts [2, 3].

Although models that were previously proposed to explain how RNA regulates PRC2 are still pending validation (reviewed at [3]), recent studies point toward an eviction model whereby RNA serves as a decoy, or a natural sink, to prevent PRC2 from interfering with active genes [4]. This model is consistent with the correlation shown between binding events of PRC2 on transcripts *in vivo* with active epigenetic marks, rather than repressive marks [4]. Furthermore, RNA inhibits the histone methyltransferase activity of PRC2 *in vitro* [5] and was shown to evict PRC2 from genes *in vivo* [6].

While observations of the interactions between PRC2 and transcripts have commonly been firm and reproducible, the RNA binding specificity of PRC2 remained puzzling and elusive. RNA immunoprecipitation approaches have collectively demonstrated that PRC2 binds thousands of transcripts *in vivo*, including both mRNAs and lncRNAs [3]. Yet, the RNA-binding activity of PRC2 is not nonspecific. Instead, PRC2 demonstrates broad binding specificity with small variations in affinity to different target transcripts, termed promiscuous binding [3, 4]. This complicated the identification of a binding motif for PRC2 within RNA, leaving an open question: how does PRC2 recognize its cognate RNA transcripts?

**Figure 1:**
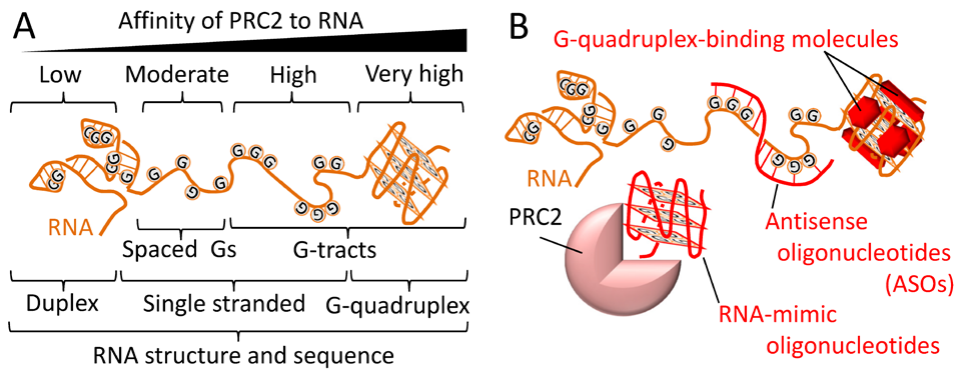
Molecular basis for the broad binding specificity (promiscuity) of PRC2 to RNA and potential ways for targeting PRC2–RNA interactions **A.** PRC2 has low affinity to double-stranded RNA and high affinity to G-tract RNAs, especially if the RNA is folded into a G-quadruplex structure. **B.** Potential strategies to inhibit RNA binding by PRC2 *in vivo*, using G-quadruplex-binding small molecules, antisense oligonucleotides or RNA mimics. Schematic representations across the figure are not to scale and include RNA in orange, guanine bases highlighted as ‘G’, agents predicted to interfere with PRC2–RNA interactions in red and PRC2 in pink.

To answer this question, we hypothesized that the PRC2-binding motif within RNA would be simple and highly abundant, given the large number of transcripts bound by PRC2. Accordingly, *in vitro* binding experiments with RNA of low-complexity sequences led to the discovery that only a few repeats of two or more consecutive guanines (G-tracts) are sufficient to substantially increase the affinity of PRC2 to RNA (Figure 1A) [7]. For example, an RNA containing ten GGAA repeats binds PRC2 *in vitro* with 50-fold higher affinity than a size-matched RNA with ten GAGA repeats. The same trend took place *in vivo*, where ectopically expressed RNA bearing ten GGAA repeats co-immunoprecipitated with PRC2 from human HEK293 cells better than a control construct, for which the repeat sequence was changed to GAGA. In addition, we showed that PRC2 binding sites within the RNA transcriptome in human cells are enriched at sequences of multiple G-tracts. Finally, PRC2 preferentially binds to RNA folded into a G-quadruplex [7]: a four-stranded structure, where G-tracts interact via Hoogsteen base pairing to form guanine quartets (Figure 1A) [8]. In contrast to this, PRC2 has low affinity to duplex RNA [7]. The abundance of G-tract sequences in the transcriptome rationalizes the broad RNA binding specificity of PRC2 *in vivo* [3]. The enrichment of G-tract sequences at Polycomb target genes provides means for RNA-mediated regulation of PRC2.

Inhibitors that specifically prevent PRC2 histone methyltranferase activity are being considered as promising anticancer therapeutics, since gain-of-function mutations in PRC2 subunits or over-expression of its subunit EZH2 are often associated with increased H3K27me3 methylation and repression of tumour suppressor genes [1]. Conversely, mutations in PRC2 subunits or its histone substrates commonly occur in certain types of leukaemia and glioma, and lead to reduced histone methyltransferase activity that is associated with derepression of oncogenes [1]. Yet, there are currently no approaches to stimulate the histone methyltransferase activity of PRC2. We propose that the enzymatic activity of PRC2 could be stimulated by disrupting the inhibitory interactions between PRC2 and its antagonistic factors, such as RNA transcripts (Figure 1B).

Our discovery of a PRC2-binding motif within RNA (Figure 1A) could now provide means for targeting PRC2–RNA interactions using readily available tools and reagents (Figure 1B). For instance, small molecules were previously developed to target G-quadruplex RNA and DNA in cells [8]. Such molecules could potentially be used to prevent PRC2 from interacting with its target transcripts. Our study has already demonstrated that G-quadruplex-binding molecules inhibit PRC2-RNA interactions *in vitro* [7]. Given the low affinity of PRC2 to duplex RNA, antisense oligonucleotides (ASO) could be used to target specific transcripts that otherwise would be bound to PRC2, and thus block PRC2-RNA interactions. Alternatively, RNA-mimic oligonucleotides could bind PRC2 to inhibit its histone methyltransferase activity. Collectively, new strategies to target the RNA-binding activity of PRC2 or its target transcripts could be used to tune the ability of PRC2 to repress polycomb-target genes and thus may broaden the toolbox available for researchers and clinicians.
